# Dual functional matrix metalloproteinase-responsive curcumin-loaded nanoparticles for tumor-targeted treatment

**DOI:** 10.1080/10717544.2019.1676843

**Published:** 2019-11-06

**Authors:** Fangyuan Guo, Qiafan Fu, Chenhao Jin, Xugang Ji, Qinying Yan, Qingliang Yang, Danjun Wu, Ying Gao, Weiyong Hong, Aiqin Li, Gensheng Yang

**Affiliations:** a College of Pharmaceutical Science, Zhejiang University of Technology, Hangzhou, China;; b Taizhou Municipal Hospital of Zhejiang Province, Taizhou, China;; c Zhejiang Share Bio-pharm Co. Ltd, Hangzhou, China

**Keywords:** Cell-penetrating peptide, cleavable peptide, curcumin, enzyme-responsive nanoparticle, targeting drug delivery

## Abstract

The limitations of anticancer drugs, including poor tumor targeting and weak uptake efficiency, are important factors affecting tumor therapy. According to characteristics of the tumor microenvironment, in this study, we aimed to synthesize matrix metalloproteinase (MMP)-responsive curcumin (Cur)-loaded nanoparticles (Cur-P-NPs) based on amphiphilic block copolymer (MePEG-peptide-PET-PCL) with MMP-cleavable peptide (GPLGIAGQ) and penetrating peptide (r9), modified to improve tumor targeting and cellular uptake. The average size of Cur-P-NPs was 176.9 nm, with a zeta potential of 8.1 mV, and they showed drug entrapment efficiency and a loading capacity of 87.07% ± 0.63% and 7.44% ± 0.16%, respectively. Furthermore, Cur release from Cur-P-NPs was sustained for 144 h at pH 7.4, and the release rate was accelerated under enzyme reaction condition. The MTT assay demonstrated that free P-NPs had favorable biosafety, and the anti-proliferative activity of Cur-P-NPs was positively correlated with Cur concentration in MCF-7 cells. Additionally, the results of cellular uptake, in vivo pharmacokinetics, and biodistribution showed that Cur-P-NPs had a good effect on cellular uptake and tumor targeting, resulting in the best bioavailability in tumor therapy. Therefore, Cur-P-NPs, as a promising drug delivery system, might lead to a new and efficient route for targeted therapy in clinical practice.

## 1. Introduction

Cancer is one of the most devastating diseases in the world, which seriously threatens the health and life of humans, and more than 10 million new cancer cases are diagnosed every year. Furthermore, the complexity of carcinogenesis limits the selectivity of treatment schemes and the efficacy of disease management, which is an urgent problem to be solved in the current international medical field (Are et al., 2013; Li & Zhang, 2019). Chemotherapy is one of the three main methods of cancer treatment. However, conventional chemotherapeutic drugs encounter the following three major challenges in clinical applications: (1) poor solubility and easy metabolism, (2) targeted selectivity and cell penetration ability, and (3) drug adverse reactions. Therefore, there is an urgent need to develop innovative technologies to promote cancer diagnosis, treatment, and prognosis.

In recent years, nanotechnology has made great progress in the fields of biomedicine, pharmacy, and drug delivery (Pandey et al., 2016; Madni et al., 2017). Nanoscale drug delivery platform can be prepared in different sizes and shapes, to improve drug solubility, in vivo stability, drug targeting, and drug toxicity (Li et al., 2013; Hu et al., 2018; Qu et al., 2018; Wang et al., 2018). However, the distribution, penetration, and release of a drug are often limited by the abnormal tumor microenvironment (TME) (Petros & DeSimone, 2010; An et al., 2014; Xu et al., 2018; Li et al., 2019). Thus, according to the TME, how to improve the bioavailability of drugs through the rational design of nanocarriers has attracted enormous research interest (Yang et al., 2018; Qiao et al., 2019).

Enzyme-response nanoparticles are an active targeted nanodelivery system based on molecular recognition between specific enzymes (overexpressed receptors) in the TME and targeting ligands modified on the surface of a drug carrier. Due to the diversity of specific enzymes in the TME, enzyme-response nanoparticles have become an excellent candidate to design intelligent drug delivery systems. It is well-known that matrix metalloproteinases (MMPs) are an indispensable conserved enzyme for extracellular matrix degradation. In normal cells, MMPs play a silent role, whereas, in tumor cells, MMPs are active and overexpressed, and play a crucial role in tumor invasion and metastasis (Kratz et al., 2001; Kessenbrock et al., 2010). Therefore, MMPs can be used as a specific target of tumor therapy. To date, a series of directed peptides (Turk et al., 2001; Shi et al., 2012; Zhu et al., 2013; Bacinello et al., 2014; Bremmer et al., 2014; Lin et al., 2014; Tanaka et al., 2015; Yu et al., 2015) has been developed as active targeting ligands for MMP action with encouraging results. However, an ideal anticancer drug carrier requires not only a selective targeting ability, but also good cellular uptake ability. Cell penetrating peptide (CPP) is an effective functional membrane ‘assistant’ (He et al., 2016). Therefore, the combination of CPP and targeted ligands is a potential strategy to achieve high selectivity and permeability of tumor cells.

Based on these facts, we selected the lipophilic drug curcumin (Cur; broad-spectrum, safe, and multidrug-resistant; Dasi et al., 2006) as the model drug. A new MMP-responsive multifunctional precursor of the carrier, MePEG-peptide-PET-PCL, was designed using the MMP-cleavable peptide (GPLGIAGQ) (Kratz et al., 2001; Shi et al., 2012) and penetrating peptide (r9) to enhance the selectivity and cell penetration of the carrier towards the targets of tumor therapy. Therefore, the tumor-targeting mechanisms and the effects of the cellular uptake of MMP-responsive curcumin (Cur)-loaded nanoparticles (Cur-P-NPs) on the breast cancer cell line, MCF-7, were analyzed in vitro and in vivo.

## 2. Materials and methods

### 2.1. Materials

Curcumin was purchased from Great Forest Biomedical Ltd. (Zhejiang, China). Peptide ((ACP)-GPLGIAGQrrrrrrrrr-(ACP)) was purchased from China Peptides Co., Ltd. (Shanghai, China). Poly(ethylene glycol) (MePEG, *M*_w_: 1900), pentaerythritol (PET), ε-caprolactone (ε-PCL), dicyclohexylcarbodiimide (DCC), succinic anhydride (CP), triethylamine (TEA), stannous 2-ethylhexanoate, 4-dimethylaminopyridine (DMAP), and dialysis membrane (*M*_w_: 8 kDa) were purchased from Aladdin (Shanghai, China). Ether, ethanol, dichloromethane, and acetonitrile were purchased from Reagent Co., Ltd. (Shanghai, China). 3-(4,5-Dimethylthiazol-2-yl)-2,5-diphenyltetrazolium bromide (MTT), trypsin, and RPMI 1640 Medium were obtained from Gibco (Merelbeke, Belgium). The human breast adenocarcinoma cell line, MCF-7, was provided by the Cell Bank of the Chinese Academy of Sciences (Beijing, China).

### 2.2. Synthesis of MePEG-peptide-PET-PCL and MePEG-PET-PCL

**Figure 1.**
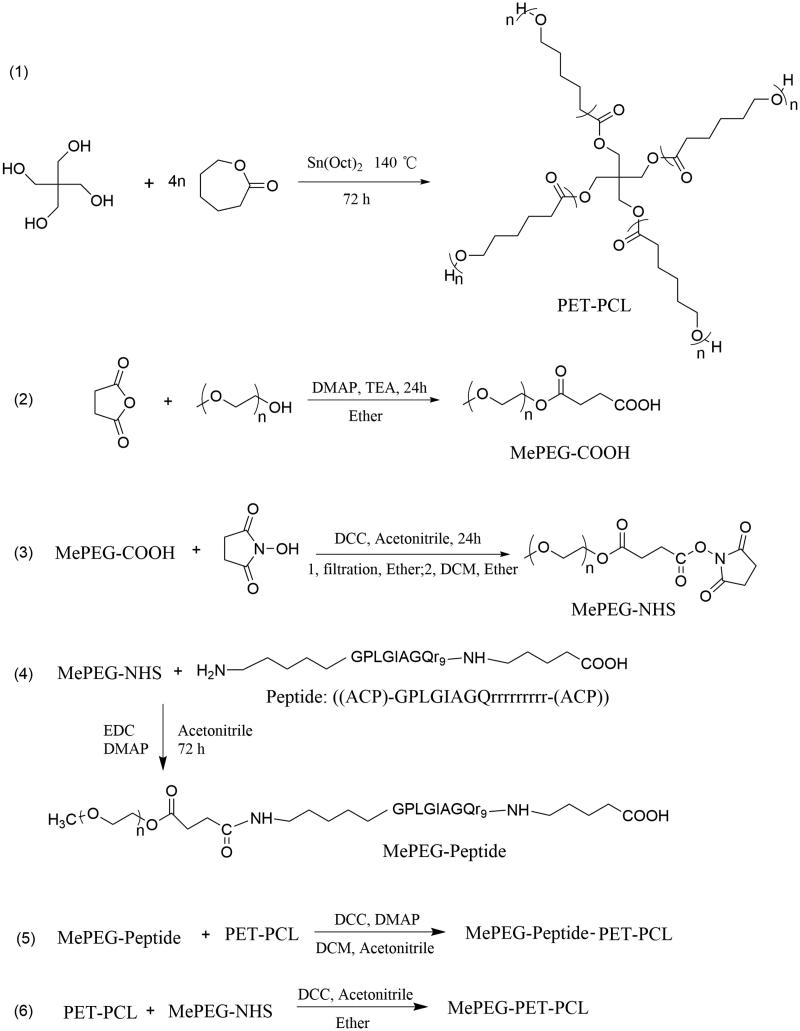
Synthesis scheme of MePEG-peptide-PET-PCL and MePEG-PET-PCL.

The synthesis schemes of MePEG-peptide-PET-PCL and MePEG-PET-PCL are shown in Figure 1.

#### 2.2.1. Synthesis of PET-PCL

PET-PCL was synthesized using the ring-opening method. Briefly, ε-caprolactone (ε-CL, 40 mmol) and pentaerythritol (PET, 1 mmol) were added into a 100-mL three-necked flask, and then heated and stirred at 140 °C for 15–20 min to produce a homogeneous mixture. Sn(Oct)_2_ (0.5% wt, catalyst) was then added and thoroughly mixed under a nitrogen atmosphere. Next, the reaction mixture was continuously stirred at 140 °C for 72 h. PET-PCL was dissolved in chloroform (∼20 mL) and precipitated into 200 mL of ice-cold methanol (chloroform/methanol = 1:10, volume ratio), and isolated by filtration twice. Finally, PET-PCL was dried under vacuum at 40 °C.

#### 2.2.2. Synthesis of MePEG-COOH

MePEG1900 (1 mmol) and succinic anhydride (1.5 mmol) were added into a 50-mL three-port flask, and then 20 mL of pyridine was added and stirred until dissolved completely. Under the protection of N_2_, DMAP (catalyst, 0.15 mmol) and TEA (acid-capturer, 0.75 mmol) were quickly added. The mixture was stirred at 25 °C for 24 h. The product (MePEG-COOH) was precipitated into 250 mL of ice-cold ether, isolated by filtration, and purified by dissolution–precipitation in ether, and then dried under vacuum.

#### 2.2.3. Synthesis of MePEG-NHS

MePEG-COOH (1 mmol) and *N*-hydroxy succinimide (1.5 mmol）were added into a 50-mL round-bottom flask. Then, acetonitrile (15 mL) was added and rapidly stirred until dissolved completely. Next, DCC (1.5 mmol) was added, and the reaction was carried out for 24 h at 25 °C. MePEG-NHS was dissolved in 15 mL of DCM and added into 250 mL of ice-cold ether. The precipitate (MePEG-NHS) was filtered and dried under vacuum.

#### 2.2.4. Synthesis of MePEG-peptide

To activate the peptide, peptide (1.278 mmol), 1-ethyl-3-(3-dimethylaminopropyl)carbodiimide hydrochloride (EDC, 8.52 mmol), and DMAP (8.52 mmol) were dissolved in 100 mL of water/acetonitrile (4:1, v/v), and stirred under dry nitrogen for 2 h in an ice-water bath. Then, MePEG-NHS (1.068 mmol) dissolved in acetonitrile (20 mL) was added, and the reaction mixture was placed at 25 °C for 48 h. The reaction mixture was then dialyzed in deionized water for 24 h to remove the organic solvent. Later, the MePEG-peptide crude product was obtained by lyophilization.

#### 2.2.5. Synthesis of MePEG-peptide-PET-PCL

MePEG-peptide (1.145 mmol), DCC (8.59 mmol), and DMAP (8.59 mmol) were dissolved in DCM (100 mL), stirred under dry nitrogen in an ice-water bath for 2 h, then PET-PCL (0.285 mmol) was added. The mixture was further stirred at 25 °C for 48 h until the reaction was completed. MePEG-peptide-PET-PCL purification was carried out by dialysis (*M*_w_: 8 kDa) and lyophilization.

#### 2.2.6. Synthesis of MePEG-PET-PCL

PET-PCL (0.25 mmol) and MePEG-NHS (1.0 mmol) were added into a 50-mL round-bottom flask, and then acetonitrile (15 mL) was added to dissolve the mixture. Thereafter, DCC (7.5 mmol) was added, and the mixture was further stirred at 25 °C for 24 h. The crude product (MePEG-PET-PCL) was precipitated from dichloromethane (DCM, 20 mL) into cold ethyl ether (1:15, v/v). The precipitate was dried in vacuo at 40 °C for 24 h.

### 2.3. Characterization of the polymers

The structures of PET-PCL, MePEG-PET-PCL, and MePEG-peptide-PET-PCL were confirmed by ^1^H-nuclear magnetic resonance (^1^H-NMR) spectroscopy (AC-III, 500 MHz; Bruker Daltonics, Billerica, MA) and Fourier transform infrared (FT-IR) spectroscopy (Nicolet 6700; Thermo Fisher Scientific, Waltham, MA). The average molecular weight (Mn), weight-average molecular weight (Mw), and polydispersity index (PDI) of the polymers were measured using the LC-20AT chromatographic instrument with a RID-10A detector by gel permeation chromatography (GPC) analysis using tetrahydrofuran as an elution solvent (flowrate: 1 mL/min, at 40 °C) and polystyrene as the standard.

### 2.4. Preparation of nanoparticles

Free peptide-modified nanoparticles (P-NPs) and Cur-P-NPs were prepared with MePEG-peptide-PET-PCL, and MePEG-peptide-PET-PCL and Cur, respectively, using a solvent evaporation method. Briefly, poloxamer (P188) surfactant (0.6 mg) was dissolved in distilled water (20 mL) as the aqueous phase. MePEG-peptide-PET-PCL (48.0 mg), and MePEG-peptide-PET-PCL (48.0 mg) and Cur (4.0 mg) were dissolved in acetone (4 mL) as a lipid phase. The lipid phase was then added dropwise into the aqueous phase under magnetic stirring. The mixture was stirred for 4 h and dried in vacuo for 1 h to remove the acetone. The obtained primary NP suspension (P-NPs and Cur-P-NPs) was passed through a 0.45-μm filter membrane to achieve a homogeneous suspension. The preparation of blank nanoparticles and Cur-loading nanoparticles (Cur-NPs) was carried out following the same method as that for P-NPs and Cur-P-NPs, respectively, using MePEG-PET-PCL, and MePEG-PET-PCL and Cur as the original material.

### 2.5. Characterization of nanoparticles

#### 2.5.1. Entrapment efficiency (EE) and drug loading (DL)

The content of Cur in Cur-P-NPs and Cur-NPs was determined by ultraviolet spectrophotometry at a detection wavelength of 420 nm. The suspensions were centrifuged at 19,000 rpm for 30 min. The precipitates (nanoparticles) were collected and lyophilized. Drug entrapment efficiency (EE) and drug-loading (DL) content were calculated as follows (Guo et al., 2017):

$$\text{EE}\;(\%)＝\frac{\left(\text{weight}\;\text{of}\;\text{Cur}\;\text{in}\;\text{Cur}-P-\text{NPs}\right)}{\left(\text{weight}\;\text{of}\;\text{total}\;\text{Cur}\right)}\times 100$$

$$\text{DL}\;(\%)＝\frac{\left(\text{weight}\;\text{of}\;\text{Cur}\;\text{in}\;\text{Cur}-P-\text{NPs}\right)}{\left(\text{weight}\;\text{of}\;\text{nanoparticle}\right)}\times 100$$

#### 2.5.2. Physicochemical properties of nanoparticles

The size distribution and zeta potential of the nanoparticles were determined at 25 °C using Malvern Zetasizer Nano ZS (Malvern Instrument Ltd., Malvern, UK). The morphology of the Cur-P-NPs was observed by transmission electron microscopy (TEM) (JEOL, JEM-1010, Tokyo, Japan) (Gratton et al., 2008).

### 2.6. *In vitro* drug release study

The Cur release rate was studied using the dialysis method at 37 °C in phosphate buffer solution (PBS) at pH 7.4. Briefly, Cur-DMSO (DMSO/H_2_O = 1/1000 (v/v), 5 mL), Cur-DMSO + 30 μ0/mL collagenase IV (5 mL), Cur-NPs (5 mL), Cur-NPs + 30 μg/mL collagenase IV (5 mL), Cur-NPs (5 mL), and Cur-P-NPs + 30 μg/mL collagenase IV (5 mL) with the same Cur content (150 μg/mL) was dialyzed (*M*_w_ 8 kDa) against 50 mL of PBS in an incubator, with shaking at 100 rpm. The external solution (5 mL) was removed and replaced with an equivalent volume of fresh dissolution medium at predetermined time points (0–144 h). The Cur content was measured per the method listed in 2.5.1. All experiments were carried out in triplicates. All data are expressed as mean ± SD.

### 2.7. Cell culture

MCF-7 cells were grown in RPMI 1640 medium supplemented with 10% (v/v) fetal bovine serum (FBS) at 37 °C with 5% CO_2_/air and 100% relative humidity. The cells were settled in 96-well plates at a density of 1 × 10^5^ cells/well in l mL of medium and allowed to attach for 24 h prior to the initiation of experiments.

### 2.8. Anti-proliferative effect of nanoparticles *in vitro*

The anti-proliferative effects of Cur-P-NPs, Cur-NPs, Cur-DMSO, P-NPs, and NPs were assessed using the MTT method. One hundred microliters of sterilized test samples or medium only (negative control, 100% cell viability) was added to cells and incubated for 48 h. All samples were prepared in triplicates. Next, 20 μL of MTT labeling reagent was added to each well, and the cells were cultured for 4 h at 37 °C. Cell viability was determined by measuring the absorbance using a microplate reader at 570 nm, with the following formula:

$$\text{The}\;\text{cell}\;\text{viability}\;(\%)={\left[A\right]}_{\text{test}}/{\left[A\right]}_{\text{control}}\times 100$$

where [A]_test_ and [A]_control_ represent the absorbance values of the test and negative control solutions, respectively. Meanwhile, the IC_50_ value of each sample was calculated using SPSS (SPSS Inc., Chicago, IL).

### 2.9. *In vitro* cellular uptake

*Qualitative analysis*: MCF-7 cells were seeded in 24-well plates (5 × 10^4^ cells/well) and treated with 200 μL of Cur-DMSO, Cur-NPs, Cur-P-NPs with 25 μg/mL Cur, or medium only (negative control) at 37 °C for 4 h. Cellular uptake was evaluated by fluorescence microscopy at 20× magnification (Eclipse Ti-S; Nikon, Tokyo, Japan).

*Quantitative analysis*: MCF-7 cells were pre-incubated in DMEM in 6-well plates (4 × 10^5^ cells/well) and treated with 2 mL of Cur-DMSO, Cur-NPs, Cur-P-NPs with 25 μg/mL Cur, or medium only (negative control) at 37 °C for 4 h. Thereafter, the medium was removed, and the cells were washed twice with PBS prior to evaluation by flow cytometry (CytoFLEX S; Beckman Coulter, Brea, CA). All experiments were carried out in triplicate.

### 2.10. Animal studies

Sprague–Dawley (SD) rats and BALB/c nude female mice were obtained from the Zhejiang Academy of Medical Sciences (Hangzhou, China) for use in the study. All experimental procedures were conducted in conformity with institutional guidelines for the care and use of laboratory animals in the Zhejiang University of Technology and conformed to the National Institutes of Health Guide for Care and Use of Laboratory Animals (Publication no. 85-23, revised 1996).

### 2.11. *In vivo* pharmacokinetics

SD rats (*n* = 15; 200 ± 20 g, female) were randomly divided into three groups. The groups were administered Cur-DMSO, Cur-NP, and Cur-P-NP by intravenous (i.v.) tail vein injection as a single dose (1.5 mg/kg Cur). Blood (0.3 mL) was sampled from the orbit after 0, 0.25, 0.5, 1, 2, 4, 8, 12, and 24 h of administration. The blood plasma was treated with acetonitrile (1 mL), vortexed for 3 min, and then centrifuged at 4 °C for 10 min at 8000 rpm. The supernatant was collected and dried using nitrogen gas at 40 °C. All samples were re-dissolved with acetonitrile (100 μL) and centrifuged at 10,000 rpm for 10 min. Next, the supernatant (20 μL) was analyzed by HPLC (Wu et al., 2019). In addition, the pharmacokinetic parameters of Cur-DMSO, Cur-NP, and Cur-P-NP were analyzed by the DAS 2.0 Practical Pharmacokinetics Program.

### 2.12. *In vivo* biodistribution study

Nude mice (15–20 g, female) were used to prepare a tumor-bearing mouse model (Guo et al., 2018). Briefly, 1 × 10^7^ MCF-7 cells in 200 μL of sterile PBS were embedded into the left armpit of the mice by subcutaneous injection. When the tumors grew to approximately 100 mm^3^ in volume, Cur-DMSO, Cur-NPs, and Cur-P-NPs with a Cur concentration of 0.20 mg/kg were administered by i.v. injection. After injection, the tumor-bearing mice were observed at 2 and 6 h using IVIS Lumina Series III (PerkinElmer, Waltham, MA). To study the tumor-targeting effect and in vivo distribution, the mice from each group were sacrificed at 24 h post-injection. The major organs (heart, liver, spleen, lung, and kidney) and the tumors were then excised and observed using IVIS Lumina Series III.

### 2.13. Statistical analysis

Student’s *t* test was used to compare the amount of drug released between Cur-NPs and Cur-P-NPs, as well as the *in vitro* anti-proliferative effect and *in vitro* cellular uptake by flow cytometry among Cur-DMSO, Cur-NPs, and Cur-P-NPs in MCF-7 cells. The results with *p* values of <.05 were considered statistically significant.

## 3. Results and discussion

### 3.1. Polymer characterization

#### 3.1.1. ^1^H-NMR

**Figure 2.**
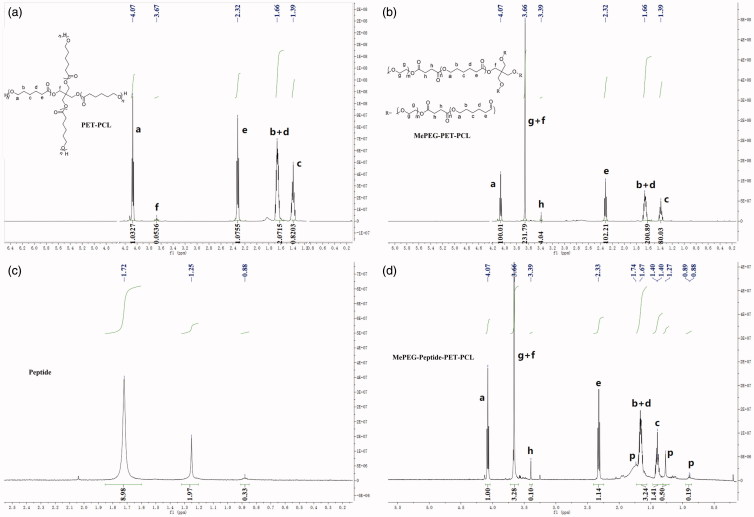
^1^H-NMR spectra of PET-PCL, MePEG-PET-PCL, peptide, and MePEG-peptide-PET-PCL.

**Figure 3.**
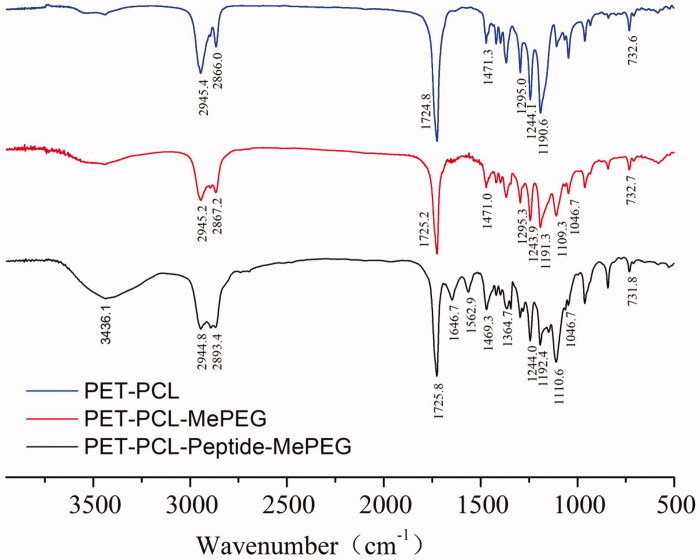
FT-IR spectra of PET-PCL, MePEG-PET-PCL, and MePEG-peptide-PET-PCL.

As illustrated in the ^1^H-NMR spectra of PET-PCL (Figure 2(a)), the chemical shifts of methylene protons (–CH_2_–) in PCL repeating units were observed at 1.39 (c), 1.66 (b + d), 2.32 (e), and 4.07 (a) ppm, while that of PET units (C(CH_2_)_4_–) was at 3.67 (f) ppm. For MePEG-PET-CL (Figure 2(b)), the peak at 3.39 (h) ppm was ascribed to the protons of the methylene group (–CH_2_–CH_2_–) in the succinic anhydride unit. Additionally, the intensity of peak at 3.66 ppm was enhanced, which was attributed to the protons (g) of the methylene group in MePEG. In the ^1^H-NMR spectrum of MePEG-peptide-PET-CL (Figure 3(d)), compared with those of the peptide (Figure 2(c)), the characteristic peaks of the peptide units were observed at 1.72, 1.25, and 0.88 ppm, while the peaks of the other moieties were the same as those of MePEG-PET-PCL.

#### 3.1.2. FT-IR

The FT-IR spectra of PET-PCL, MePEG-PET-PCL, and MePEG-peptide-PET-PCL are presented in Figure 3. Compared with those of PET-PCL and MePEG-PET-PCL, the peaks at 1724.8 and 1244.1 and 1190.6 cm^−1^ corresponded to C=O bond stretching and C–O vibration bond in ester linkage, respectively, while the peaks at 2945.4, 2866.0, 1471.3, and 732.6 cm^−1^ were attributed to the characteristic peaks of C–H in the methylene group on the PCL and MePEG fragments. The peaks at 1109.3 and 1046.7 cm^−1^ were attributed to the characteristic C–O–C stretching vibration in MePEG. For MePEG-peptide-PET-PCL, besides the peaks of MePEG-PET-PCL, the signals at 3436.1, 1646.7, 1364.7, and 1562.9 cm^−1^ corresponded to the vibrations of O–H, C=O, C–N, and N–H bonds of amido bond in the peptide, respectively. According to the hydrogen spectrum and infrared analysis results, MePEG-PET-PCL and MePEG-peptide-PET-PCL were successfully synthesized.

#### 3.1.3. GPC

The GPC results are presented in Table 1. The Mn of PET-PCL, MePEG-peptide, and MePEG-peptide-PET-CL was 16,517.3, 18,850.6, and 19,953.8 Da, respectively. The PDI of each copolymer was approximately 1.01–1.13 (<1.20), showing a narrow distribution, thereby indicating proper quality for the subsequent formation of iso-dispersed NPs.

**Table 1.** Mn, Mw, and PDI of copolymers.

Copolymer	*M*_n_ (Da)	*M*_w_ (Da)	PDI
PET-PCL	16,517.3	16,600.1	1.01
MePEG-PET-PCL	18,850.6	19,276.1	1.02
MePEG-peptide-PET-PCL	19,953.8	22,494.2	1.13

### 3.2. Characterization of nanoparticles

**Figure 4.**
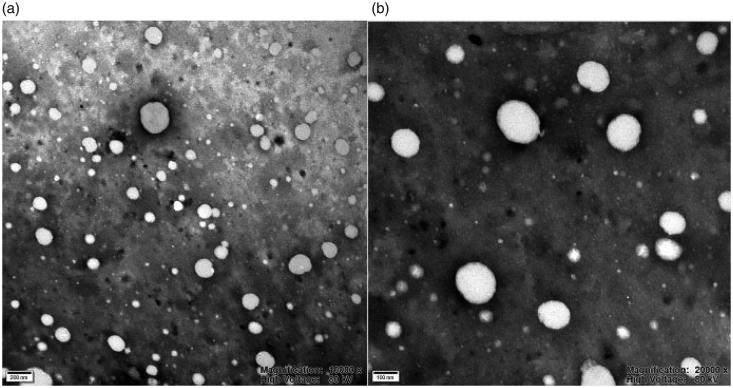
TEM photograph of Cur-P-NPs. (a) 10,000 × magnification; (b) 20,000 × magnification.

The particle size, zeta potential, and EE and DL values of nanoparticles are listed in Table 2. The average diameter of P-NPs, Cur-NPs, and Cur-P-NPs was 152.7, 142.9, and 176.9 nm with PDI of 0.101, 0.088, and 0.116, respectively, indicating a narrow size distribution for all nanoparticles. The zeta potential of Cur-NPs was electronegative (−14.6 ± 0.6 mV). For P-NPs and Cur-P-NPs, the zeta potentials were electropositive (ranged from 6.9 to 8.1 mV) due to the modification of the peptide (r9 containing a strong positive charge), while the positively charged corona could improve the effective cellular uptake (Guo et al., 2018). Moreover, compared with those of Cur-NPs, the EE and DL values of Cur-P-NPs decreased to 87.07% and 7.44%, respectively, owing to the hydrophilia enhancement. Further comparison of the linear polyester nanoparticle (reported in our previous work) based on the MePEG-peptide-PCL (Guo et al., 2018) showed that the DL values were between 7.44 and 7.58%, showing extreme similarity. However, the Cur-P-NPs showed better encapsulation efficiency (87.07%) than that of the linear polyester (MePEG-peptide-PCL) nanoparticles (80.12%). This phenomenon was produced from the characteristics of multi-branched structures. The branched chains have stronger steric resistance, which caused the Cur-P-NPs to gain a more stable spatial structure, thereby improving the efficiency of drug encapsulation. Additionally, the TEM analysis demonstrated that Cur-P-NPs were nearly spherical with the mean size of around 170 nm (Figure 4). This result is in accordance with the dynamic light scattering analysis (Table 2).

**Table 2.** Characterization of nanoparticles.

Sample	Particle size	PDI	Zeta	EE	DL
	(nm)		(mV)	(%)	(%)
P-NPs	152.7 ± 0.5	0.101 ± 0.014	6.9 ± 0.6	–	–
Cur-NPs	143.9 ± 0.9	0.088 ± 0.024	−14.6 ± 0.6	94.37 ± 1.36	9.65 ± 0.32
Cur-P-NPs	176.9 ± 0.5	0.116 ± 0.037	8.1 ± 0.7	87.07 ± 0.63	7.44 ± 0.16

### 3.3. *In vitro* drug release study

The *in vitro* drug release study was performed at 37 °C at pH 7.4 corresponding to the environment of blood, while collagenase IV (containing MMP-2/9) (Shi et al., 2012) was used to further study drug release under the TME.

**Figure 5.**
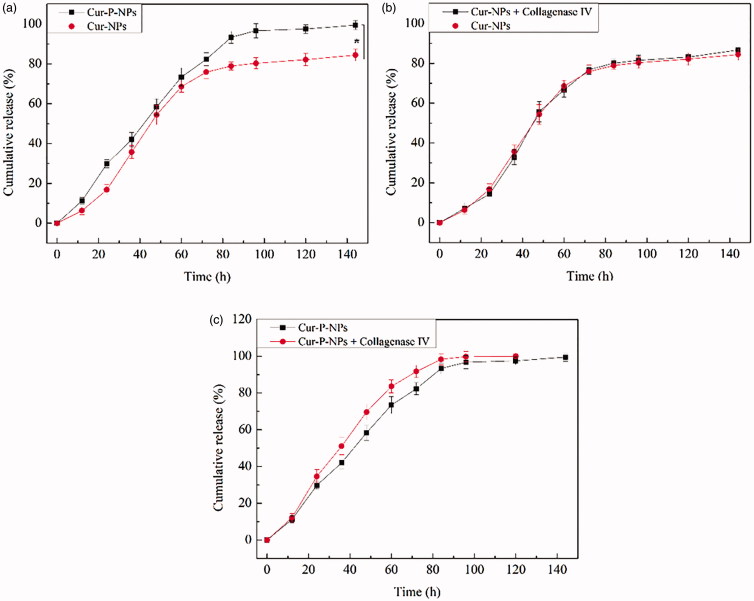
Release curve of (a) Cur-NPs and Cur-P-NPs; (b) Cur-NPs and Cur-NPs + collagenase IV; (c) Cur-P-NPs and Cur-P-NPs + collagenase IV (*p < .05).

Figure 5(a) shows the results of Cur release from Cur-NPs and Cur-P-NPs under blood-simulated environment. The curves of the Cur-NPs and Cur-P-NPs exhibited a sustained Cur release profile, and the process could be divided into two stages. Initially, a fast and stable release rate of Cur was monitored. During this stage (0–84 h), Cur enters the releasing medium by diffusion because of the difference between internal and external concentrations. The Cur-P-NPs release curve displayed a near-constant release rate of 1.11% h^−1^ with an accumulation release rate of 93.3%, whereas, the average release rate of Cur-NPs was 0.939% h^−1^ and the total released was approximately 78.9%. By comparing Cur-NPs and Cur-P-NPs, Cur-P-NPs exhibited a faster release rate than that of Cur-NPs. This result suggests that Cur from Cur-P-NPs easily diffused into the medium. According to the self-assembled regulation, Cur-NPs and Cur-P-NPs were formed with a hydrophilic shell (MePEG or MePEG-peptide segment) and a hydrophobic core (PET-PCL segment). Owing to the existence of the peptide, Cur-P-NPs had stronger hydrophilicity than did Cur-NPs, which allowed the release medium to diffuse into the nanoparticle easier and induced a faster release rate. Second, the process came into a slow release rate. During this stage (84–144 h), Cur from the Cur-P-NPs was completely released, whereas the accumulation release rate of Cur-NPs was 84.44%. However, owing to a more difficult diffusion deep into the hydrophobic core of the nanoparticle and the decreasing difference between internal and external concentrations, the release rate decreased, with only 6.7% and 5.5% of Cur released from Cur-P-NPs and Cur-NPs, respectively.

Figure 5(B,C) shows the results of Cur release from the Cur-NPs and Cur-P-NPs, respectively, under the tumor-simulated microenvironment. In Figure 5(b), the release curves between Cur-NPs and Cur-NPs + collagenase IV were barely changed, indicating that collagenase IV had a negligible effect on Cur release from Cur-NPs. In Figure 5(c), the Cur-P-NPs + collagenase IV group displayed a faster Cur release rate than that of only Cur-P-NPs, suggesting that the structure of Cur-P-NPs could be modified by collagenase IV(MePEG-cleavable peptide was removed), which could create more diffusion channels inside the nanoparticle and allow Cur to diffuse more easily into the medium.

### 3.4. *In vitro* anti-proliferative assay

**Figure 6.**
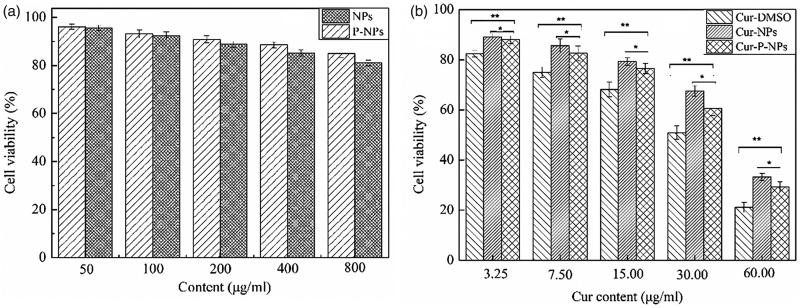
*In vitro* anti-proliferative assay of (a) the blank nanoparticles (b) Cur-loaded carriers (**p < .01, *p < .05).

Figure 6(a,b) summarizes the anti-proliferative effect of blank nanoparticles and Cur-loaded nanoparticles against MCF-7 cells, respectively. In Figure 6(a), for both NPs and P-NPs, cell viability decreased as the nanoparticle content increased, compared with that of each other, NPs showed a marginally better anti-proliferative ability than P-NPs. However, both NPs and P-NPs showed favorable biosafety, with cell viabilities ranging from 96.06% to 81.05% when co-cultured at different concentrations of 50, 100, 200, 400, and 800 μg/mL. In Figure 6(b), all Cur-loaded carriers displayed a Cur dose-dependent anticancer activity. The cell viabilities of 89.11–21.16% were measured after 48 h of incubation. Meanwhile, the anti-proliferative ability decreased as follows: Cur-DMSO > Cur-P-NPs > Cur-NPs. Owing to the favorable biosafety of NPs and P-NPs, the released Cur was considered the main element inhibiting cell growth. Thus, Cur-DMSO displayed the best anticancer activity. Moreover, Cur in Cur-P-NPs had a faster release rate than that of Cur-NPs, resulting in Cur-P-NPs having higher anticancer activity than that of Cur-NPs. Additionally, the IC_50_ values of Cur-DMSO, Cur-NPs, and Cur-P-NPs for MCF-7 cells were 24.11, 43.63, and 35.65 μg/mL, respectively.

### 3.5. *In vitro* cellular uptake

**Figure 7.**
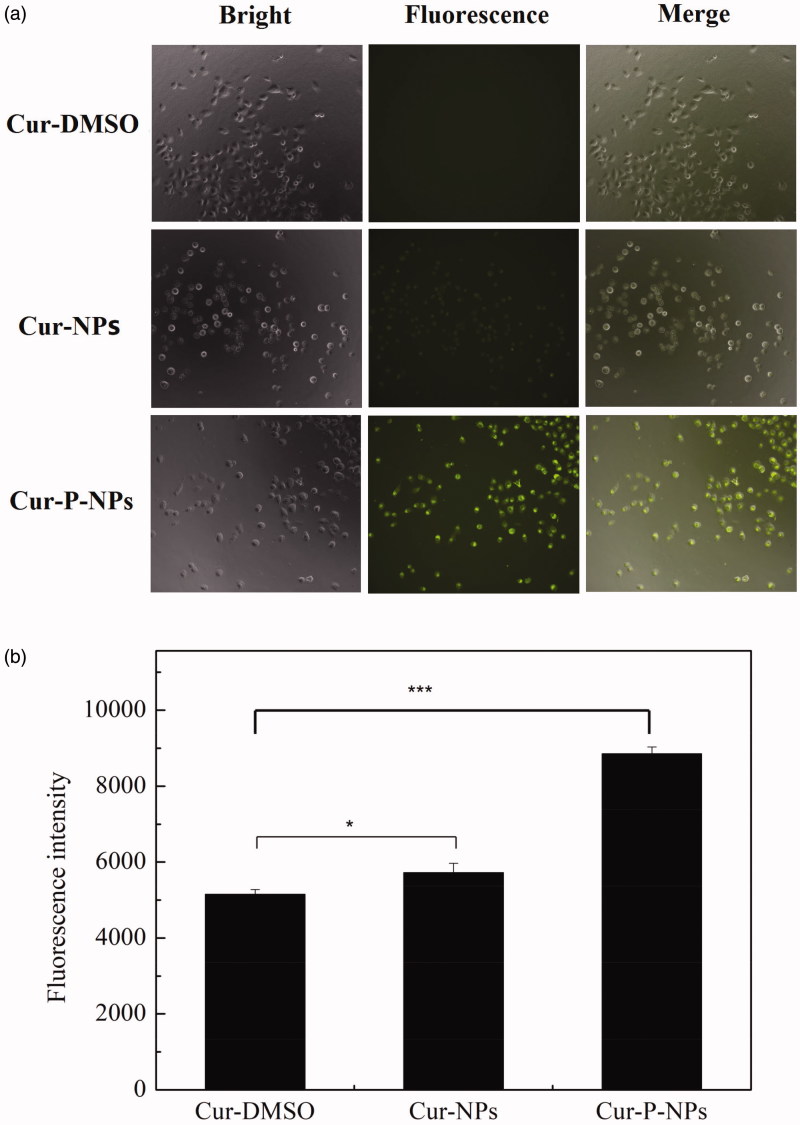
Cellular uptake in MCF-7 cells. (a) Fluorescence microscopy images of Cur-DMSO, Cur-NPs, and Cur-P-NPs (20 × magnification). (b) Flow cytometry analysis of Cur-DMSO, Cur-NPs, and Cur-P-NPs (***p < .001, *p < .05).

Figure 7(a,b) shows the results of in vitro cellular uptake of Cur-DMSO, Cur-NPs, and Cur-P-NPs by MCF-7 cells. For Figure 7(a) (fluorescence microscopy images), there was almost no fluorescence associated with Cur-DMSO, indicating the very weak ability of permeability. Due to the excellent biocompatibility of nanoparticles (Guo et al., 2017), Cur-NPs and Cur-P-NPs exerted better internalization (with a stronger fluorescence intensity) by MCF-7 cells than did Cur-DMSO. Furthermore, compared with that of Cur-NPs and Cur-P-NPs, due to the cellular uptake inhibition of PEGylation, the fluorescence intensity of Cur-NPs was weak. Inversely, the fluorescence intensity of Cur-P-NPs was noticeably strong. Besides the excellent ability of r9 (CPP) in the peptide (GPLGIAG-r9) to enhance the cellular uptake, the other reasonable explanation is that MePEG-GPLGIAGQ-r9 might be fragmented to MePEG-GPLGIAGQ and r9 by the MMP enzyme, with the separation of MePEG-GPLGIAGQ, leaving only r9 together with NPs. Therefore, the cellular uptake ability was further improved by the synergistic effect (Guo et al., 2018). As shown in Figure 7(b) (flow cytometry analysis), the means of the fluorescence intensity of Cur-DMSO, Cur-NPs, and Cur-P-NPs groups were 5148.7, 5718.3, and 8851.3, respectively. By comparison, the fluorescence intensity of the Cur-NPs and Cur-P-NPs groups were 11.1% and 71.9% enhanced when compared to Cur-DMSO, respectively. These results are consistent with the results of the qualitative analysis by fluorescence microscopy.

**Figure 8.**
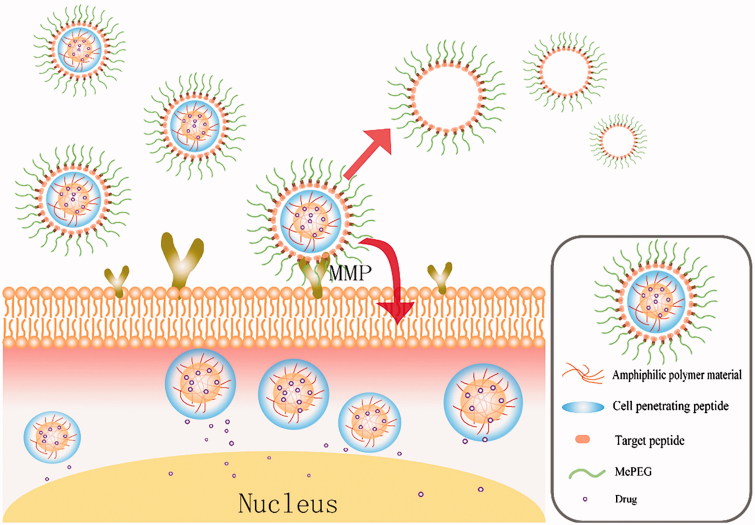
Detailed schematic of Cur-P-NPs *in vivo* delivery.

The detailed schematic of Cur-P-NPs *in vivo* delivery is shown in Figure 8.

### 3.6. *In vivo* pharmacokinetic study

**Figure 9.**
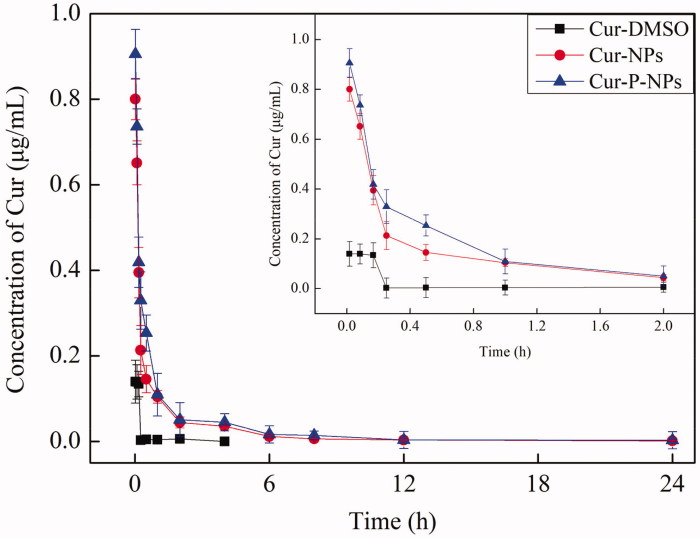
Concentration of Cur in plasma versus time after intravenous injection of Cur-DMSO, Cur-NP, and Cur-P-NP solution.

The change in plasma concentration of a drug with time is one of the important aspects of pharmacokinetics. The profiles of Cur concentration in plasma at 0.0167–24 h are shown in Figure 9. The Cur-DMSO group had the fastest elimination rate *in vivo*. There was only 0.1397 μg/mL of Cur measured in 1 min (0.0167 h) after tail vein injection, while nearly 100% of the Cur was eliminated after 2 h. For Cur-NPs and Cur-P-NPs, the curves of Cur elimination were almost consistent with each other. In both cases, Cur was rapidly eliminated as the Cur concentration was decreased from 0.9053 to 0.1044 μg/mL in the first 1 h, followed by a slow decrease period (1–24 h) as the Cur concentration was decreased from 0.1044 to 0.0014 μg/mL. Compared with those of Cur-DMSO, Cur-NPs, and Cur-P-NPs, the nanoparticle groups showed increased Cur concentration and long elimination time *in vivo*, suggesting that nanoparticle formulations, as carriers, could enhance the internal circulation time, and further improve the bioavailability of Cur (Guo et al., 2017).

In addition, pharmacokinetic parameters, such as the area under the drug concentration-time curve values (AUC_(0-∞)_), biological half-life (*t*_1/2_), maximal peak concentration (*C*_max_), and total body clearance (Cl) of carriers are listed in Table 3. At the same dose of Cur, the AUC_(0−∞)_ value of the Cur-P-NPs group (0.6431 ± 0.0504 μg/mL/h) was 1.35-fold greater than that of the Cur-NPs group and 21.08-fold greater than that of the Cur-DMSO group. The *C*_max_ value of Cur-P-NPs (0.9053 ± 0.0894 μg/mL) was 1.13-fold higher than that of the Cur-NPs group and 6.48-fold higher than that of the Cur-DMSO group. The *t*_1/2_ value of Cur-P-NPs (0.1229 ± 0.0457 h) was 1.42-fold higher than that of the Cur-NP group and 1.65-fold higher than that of the Cur-DMSO group. The Cl value of Cur-P-NP (3248 ± 288 mL/h/kg) was the lowest, only 0.69- and 0.089-folds lower than that of Cur-NPs and Cur-DMSO, respectively. As we know, larger AUC_(0−∞)_, higher *C*_max_, longer *t*_1/2_ and Cl values induce greater bioavailability. Overall, Cur encapsulated in nanoparticles not only improved the bio-stability but also prolonged the circulation time *in vivo*. Thereby, Cur-NPs and Cur-P-NPs showed much greater bioavailability than did Cur-DMSO. Additionally, compared with those of Cur-NPs and Cur-P-NPs, owing to a better cellular uptake, Cur-P-NPs could be more easily internalized by tumors, which acted as a drug reservoir by way of the sustained release of Cur, leading to the better bioavailability of Cur-P-NPs.

**Table 3.** Pharmacokinetic parameters of Cur-DMSO, Cur-NPs, and Cur-P-NPs (*n* = 6).

Parameter	Cur-DMSO	Cur-NPs	Cur-P-NPs
AUC_(0–∞)_ (μg/mL × h)	0.0305 ± 0.0014	0.4749 ± 0.0398	0.6431 ± 0.0504
*C*_max_ (μg/mL)	0.1397 ± 0.0579	0.8002 ± 0.0775	0.9053 ± 0.0894
*t*_1/2_ (h)	0.0743 ± 0.0349	0.0863 ± 0.0402	0.1229 ± 0.0457
Cl (mL/h/kg)	3.618 × 10^4^±563	4856 ± 354	3248 ± 288

### 3.7. *In vivo* biodistribution studies

**Figure 10.**
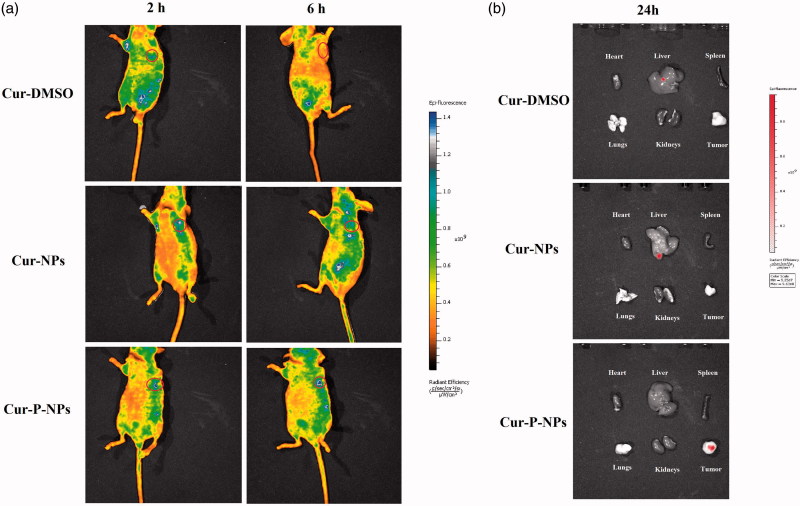
Biodistribution imaging of Cur-loaded carriers in MCF-7-inoculated athymic nude mice after i.v. injection. (a) The whole body and (b) the main organs.

To further study the targeting effect of MMP-responsive nanoparticles (Cur-P-NPs), fluorescence images of Cur-DMSO and Cur-NPs (as the control groups) and Cur-P-NPs in MCF-7 xenograft-bearing nude mice at 2 and 6 h post-injection (Figure 10(a)) or in the excised heart, liver, spleen, lungs, kidneys, and tumors at 24 h post-injection (Figure 10(b)) were obtained. As shown in Figure 10(a), in the Cur-DMSO group, most of the Cur was enriched in other organs, and only a slight fluorescence signal was detected in the tumor at 2 h post-injection. However, the fluorescence signal in tumor disappeared, and the total fluorescence intensity was quickly reduced at 6 h post-injection, indicating poor targeting selection and rapid metabolism of Cur-DMSO in vivo. For Cur-NPs, the fluorescence signals mainly appeared surrounding the right forelimb position and tumor at 2 h post-injection. It displayed stronger fluorescence intensity than that of Cur-DMSO in tumor due to the passive targeting ability of nanoparticles (the EPR effect). However, after 6 h of injection, the fluorescence intensity in tumor was decreased, and the fluorescence signals in other tissues were significantly increased, suggesting that the EPR effect cannot enrich the nanoparticles in tumor durably and efficiently. With time, Cur-NPs were captured or metabolized by other tissues due to inefficient cellular uptake, which significantly decreases the passive targeting effect. Whereas, for Cur-P-NPs, the brightest fluorescence was observed in the tumor at 2 h post-injection, which demonstrated that the best tumor-targeting selection was carried out under the synergistic effect of passive targeting (the EPR effect) and active targeting (the combination between ligands (cleavable peptide) and MMPs). Additionally, after 6 h of injection, most of the Cur was still accumulated in the tumor, while the fluorescence intensity was not reduced because of the efficient cellular uptake and sustained Cur release profile of Cur-P-NPs. Furthermore, the results of the *ex vivo* tissue images (Figure 10(b)) showed that Cur in Cur-P-NPs was still accumulated in tumor, while Cur in Cur-DMSO and Cur-NPs was observed in the liver (the main metabolic organ) at 24 h post-injection, suggesting the best tumor-targeting selection and bioavailability, which was consistent with the results obtained for *in vivo* biodistribution.

To date, a novel strategy for the tumor-targeted delivery of Cur with MMP response in the tumor microenvironment has been prepared, with proper targeting and bioavailability for tumor therapy. However, the full cure effects of the system in an animal model were not evaluated. Therefore, to further understand the anticancer mechanism of this system, *in vivo*, the pharmacodynamics and detail drug delivery process should be examined in future studies.

## 4. Conclusions

In conclusion, a novel MMP-responsive amphiphilic block copolymer, MePEG-peptide-PET-PCL, was successfully synthesized and applied in anticancer drug delivery. The dual-functional Cur-loaded nanoparticles (Cur-P-NPs) with uniform particle size, narrow size distribution, and ideal EE and DL values were obtained by self-assembling. The *in vitro* evaluation demonstrated that Cur release from Cur-P-NPs was a sustained drug release process, and the release rate could be accelerated by collagenase IV. Biosafety and Cur dose-dependent anticancer activity in MCF-7 cells were measured using free P-NPs and Cur-P-NPs, respectively. Additionally, Cur-P-NPs exerted the best effect on cellular uptake due to the synergistic effect of the CPP and cleavable peptide. *In vivo* pharmacokinetics showed that Cur-P-NPs had a stronger targeting ability to MCF-7 xenografts than to normal tissue, leading to considerably increased bioavailability. Overall, this study highlights the strong potential of MMP-responsive nanoparticles to enhance the accumulation and cellular uptake ability of lipid-soluble drugs into tumors, which is a promising system in clinical settings with targeting selection and high bioavailability for cancer treatment.
